# Genomics, Transcriptomics, and Metabolomics Reveal That Minimal Modifications in the Host Are Crucial for the Compensatory Evolution of ColE1-Like Plasmids

**DOI:** 10.1128/msphere.00184-22

**Published:** 2022-11-23

**Authors:** Manuel Ares-Arroyo, Miguel Fernández-García, Emilia Wedel, Natalia Montero, Coral Barbas, M. Fernanda Rey-Stolle, Antonia Garcia, Bruno González-Zorn

**Affiliations:** a Antimicrobial Resistance Unit (ARU), Departamento de Sanidad Animal and Centro de Vigilancia Sanitaria Veterinaria (VISAVET), Complutense University of Madrid, Madrid, Spain; b Center for Metabolomics and Bioanalysis (CEMBIO), Facultad de Farmacia, Universidad San Pablo-CEU, CEU Universities, Urbanización Montepríncipe, Boadilla del Monte, Spain; JMI Laboratories

**Keywords:** plasmid evolution, plasmid compensation, antimicrobial resistance, compensatory evolution

## Abstract

Plasmid-mediated antimicrobial resistance is one of the major threats to public health worldwide. The mechanisms involved in the plasmid/host coadaptation are still poorly characterized, and their understanding is crucial to comprehend the genesis and evolution of multidrug-resistant bacteria. With this purpose, we designed an experimental evolution using Haemophilus influenzae RdKW20 as the model strain carrying the ColE1-like plasmid pB1000. Five H. influenzae populations adapted previously to the culture conditions were transformed with pB1000 and subsequently evolved to compensate for the plasmid-associated fitness cost. Afterward, we performed an integrative multiomic analysis combining genomics, transcriptomics, and metabolomics to explore the molecular mechanisms involved in the compensatory evolution of the plasmid. Our results demonstrate that minimal modifications in the host are responsible for plasmid adaptation. Among all of them, the most enriched process was amino acid metabolism, especially those pathways related to serine, tryptophan, and arginine, eventually related to the genesis and resolution of plasmid dimers. Additional rearrangements occurred during the plasmid adaptation, such as an overexpression of the ribonucleotide reductases and metabolic modifications within specific membrane phospholipids. All these findings demonstrate that the plasmid compensation occurs through the combination of diverse host-mediated mechanisms, of which some are beyond genomic and transcriptomic modifications.

**IMPORTANCE** The ability of bacteria to horizontally transfer genetic material has turned antimicrobial resistance into one of the major sanitary crises of the 21st century. Plasmid conjugation is considered the main mechanism responsible for the mobilization of resistance genes, and its understanding is crucial to tackle this crisis. It is generally accepted that the acquisition and maintenance of mobile genetic elements entail a fitness cost to its host, which is susceptible to be alleviated through a coadaptation process or compensatory evolution. Notwithstanding, despite recent major efforts, the underlying mechanisms involved in this adaptation remain poorly characterized. Analyzing the plasmid/host coadaptation from a multiomic perspective sheds light on the physiological processes involved in the compensation, providing a new understanding on the genesis and evolution of plasmid-mediated antimicrobial-resistant bacteria.

## INTRODUCTION

Antimicrobial resistance (AMR) is a dramatically growing threat to public health worldwide, causing hundreds of thousands of deaths every year (1). Mobile genetic elements (MGEs) play a central role in this sanitary challenge, as they propagate AMR genes between bacteria through horizontal gene transfer (2). Among all MGEs, plasmids are considered to be the main spreaders of AMR in clinical environments (3); hence, understanding their ecology and evolution is urgent (4).

The massive ubiquitousness of plasmids is intriguing, as plasmids usually impose a fitness cost to their hosts, hindering plasmid-carrying bacteria in their niches when no positive selection acts in benefit of the replicon (5). The causes of the plasmid-associated fitness cost are extremely variable; nonexclusive; and dependent on the plasmid, the host, and the environment (6). Yet, many authors have attempted to understand its source and have provided recent reviews (7, 8). In general terms, most of the fitness cost is produced by the expression of the plasmid-borne genes, as this process is highly energetically demanding and involves the sequestration of numerous resources of the bacteria (9).

In turn, plasmids show diverse strategies to avoid the purifying selection triggered by their fitness cost, such as high conjugational rates or piggybacking niche adaptation (10, 11). Notwithstanding, in recent years, it has been demonstrated that a major factor involved in plasmid persistence is compensatory evolution, a plasmid/host coadaptation process through which the plasmid fitness cost is alleviated over time (12–15). Its mechanisms are diverse and, in some cases, suitable for different co-occurring plasmids (16, 17). Some examples include adjusting the altered transcriptional demand produced by the plasmid (18), modifying the conjugational rates (14), gaining (19) or losing (20, 21) plasmid-borne genes, disrupting cytotoxic plasmid-encoded proteins (22), or modifying plasmid- or chromosome-encoded proteins that interfere with the physiology of the bacterium, mostly helicases (16, 22, 23).

In most of the published work, the molecular basis of the compensatory evolution occurred in mutations located in the chromosome and/or the plasmid (18, 21, 23–28). However, in many other studies, the molecular basis remained uncharacterized, either because it was not the aim of the study or because no responsible mutation was identified (12, 14, 17, 29, 30). Furthermore, only a few studies have analyzed the transcriptional modifications during plasmid compensation (18, 23, 31) believed to play a key role even in the absence of mutations (32).

There is a notable lack of knowledge regarding nongenomic factors involved in the plasmid-host coadaptation (9). Thus, the aim of our work is to investigate the fitness cost imposed by a wild-type ColE1-like plasmid and the strategies of its subsequent compensation, using an integrative multiomic approach. As a model, we used the strain Haemophilus influenzae RdKW20 (33) and the plasmid pB1000 (34). To our knowledge, this is the first time that genomics, transcriptomics, and metabolomics have been combined with experimental evolution to analyze in a complementary way the mechanisms involved in the compensatory evolution of a plasmid. This work provides new insights into the plasmid-host coadaptation process, a key driver of bacterial evolution, of great interest in the fight against antimicrobial resistance.

## RESULTS

### The fitness cost imposed by pB1000 is compensated over 100 generations.

We designed a five-replicate evolutionary experiment detailed in Materials and Methods. Briefly, five H. influenzae RdKW20 populations (hereafter “*Rd-Pre*”) were evolved for 100 generations to adapt to the culture conditions. Then, adapted populations (“*Rd-T0*”) were transformed with pB1000 (“*Rd/pB-T0*”). Afterward, both plasmid-free and plasmid-bearing bacteria were evolved for 100 generations resulting in the populations “*Rd-T100*” and “*Rd/pB-T100*,” respectively.

**FIG 1:**
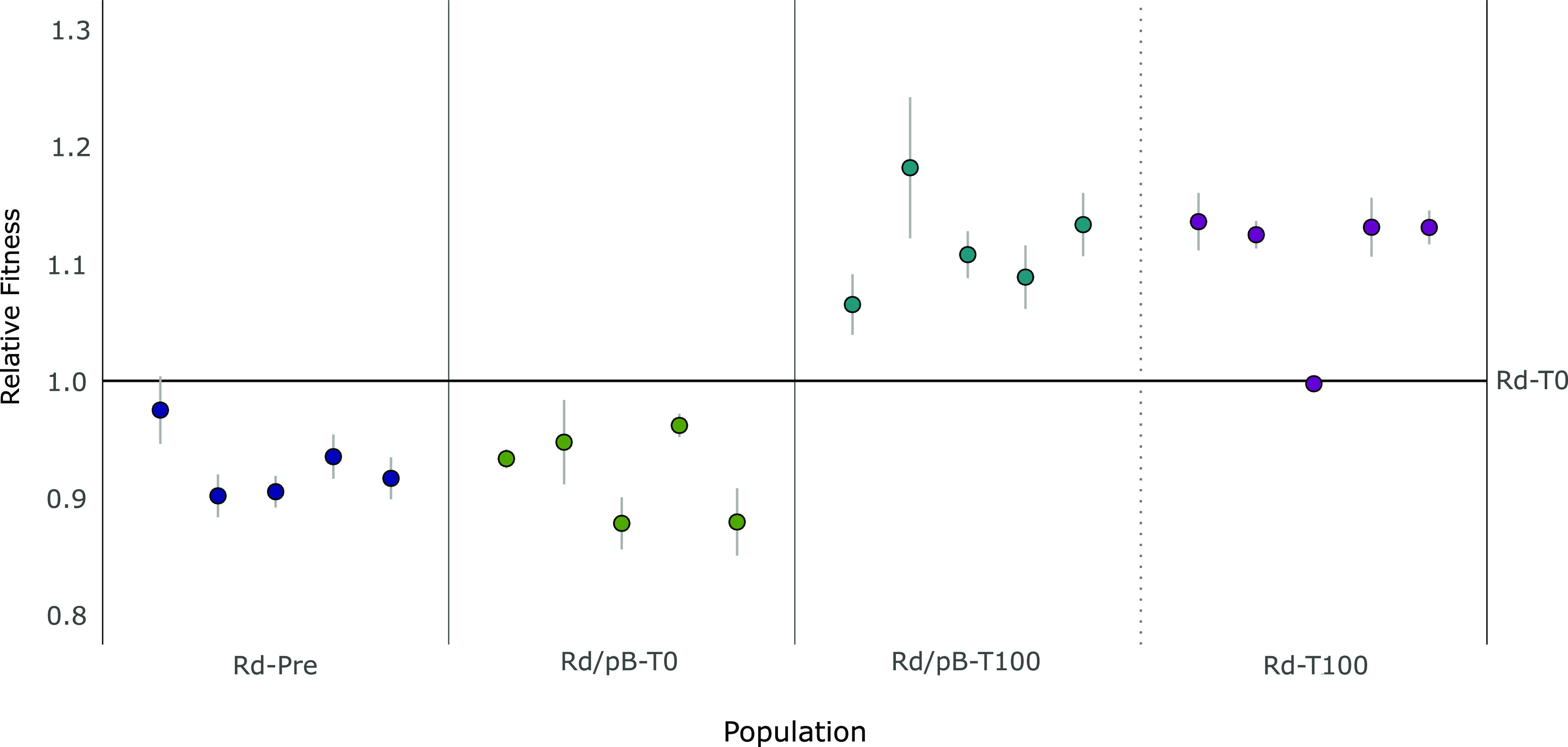
Relative fitness during the experimental evolution. Each dot represents the mean of three competition experiments, with vertical lines showing the standard error of the mean. The following populations are represented by a color: Rd-Pre, bacteria prior to the adaptation to the media (dark blue); Rd/pB-T0, bacteria already adapted to the media that had recently acquired the plasmid (green); Rd/pB-T100, bacteria that have evolved with the plasmid (turquoise); and Rd-T100, evolved populations without the replicon (purple). For the representation, the relative fitness of each population, determined through different competition experiments (Rd-Pre versus known Rd/pB1000, Rd/pB-T0 and Rd/pB-T100 versus Rd-T0, and Rd-T100 versus Rd/pB-T100), has been standardized taking as a reference the relative fitness of the adapted populations without the plasmid (Rd/T0).

First, we measured the initial adaptation to the culture conditions through competition experiments between Rd-Pre and Rd-T0 against a previously described pB1000-bearing H. influenzae RdKW20 strain (35). The coefficient of selection (*s*) of our five evolution lines increased from 0.03 ± 0.01 at the starting point to 0.16 ± 0.01 in the Rd-T0 populations (*t* = 17.71, *P* < 0.05), confirming a higher adaptation to the media. Then, we analyzed how the plasmid acquisition and coevolution affected the relative fitness of the populations. The recent acquisition of pB1000 imposed a fitness cost of 5.93% (*s* = −0.0593 ± 0.01) (Fig. 1). After 100 generations maintaining pB1000, bacteria showed a dramatic increase in their fitness (*t* = 7.36, *P* < 0.05), compensating the initial burden imposed by pB1000 and reaching a relative fitness 15.91% higher than the ancestral Rd-T0 (*s *= 0.159 ± 0.04). Lastly, we verified that the fitness cost had been successfully compensated by competing the evolved plasmid-bearing against the evolved plasmid-free populations (*s *= 0.0204 ± 0.01). The results suggest that additional mechanisms have been involved in a later adaptation during the evolution, as both Rd/pB-T100 and Rd-T100 have further improved their relative fitness compared with Rd-T0 (Fig. 1).

### No modifications in pB1000 are responsible for its adaptation.

**FIG 2:**
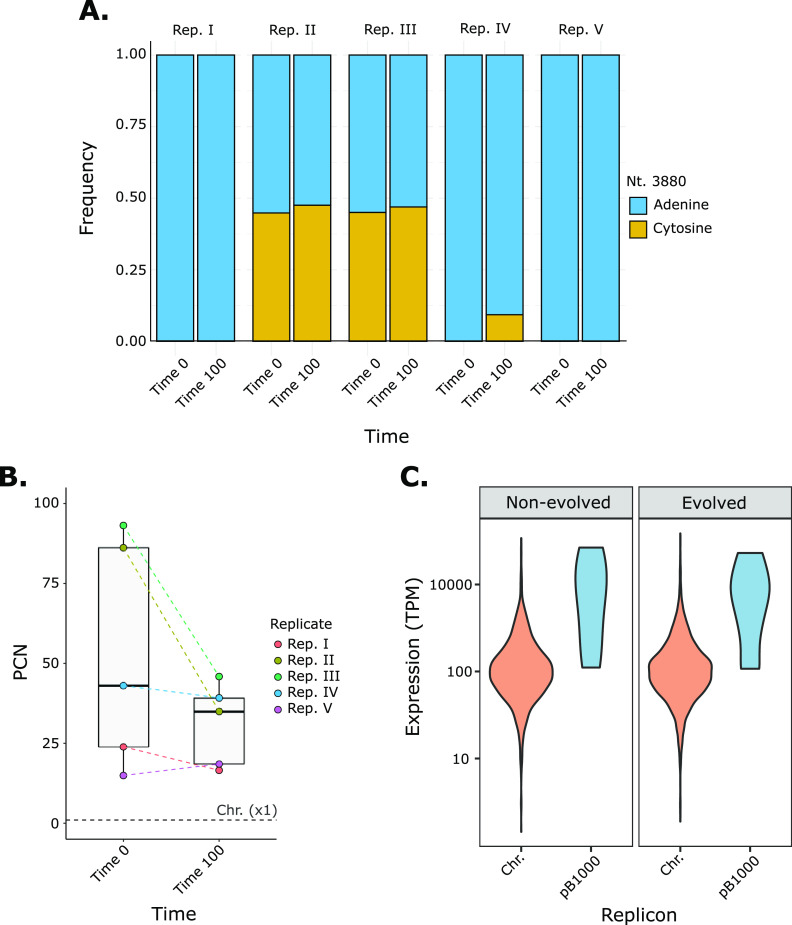
Dynamics of pB1000 during the experimental evolution. (A) pB1000 heteroplasmy. Frequency of each allele of the nucleotide 3880 in pB1000. Bars are grouped according to the evolutionary replicate (I, II, III, IV, and V). The *x* axis represents the evolution time point (“Time 0,” nonevolved; “Time 100,” evolved), whereas the *y* axis represents the frequency of the SNP (from 0 to 1). (B) Plasmid copy number. PCN of pB1000 before (“Time 0” in *x* axis) and after (“Time 100” in *x* axis) the evolution. The colored dashed lines between points connect the PCN of the same evolutionary line. The gray horizontal dashed line represents the copy number of the Chromosome (Chr.), estimated as 1. (C) Transcriptional demand of pB1000. The transcriptional demand of the chromosome (“Chr.” in *x* axis, orange) and the plasmid (“pB1000” in *x* axis, blue) is represented as the transcripts per kilobase million (TPM) of each one of their genes, shown as the average of all the population bearing pB1000, which are both evolved (Rd/pB-T0) and nonevolved (Rd/pB-T100).

Once the plasmid compensation was verified, we analyzed the genomic and transcriptomic modifications of the plasmid. First, we examined the sequence of pB1000 to determine the presence of compensatory mutations. Although no plasmid mutations were fixed during the evolution, we observed the same single nucleotide polymorphism (SNP) causing a heteroplasmy genotype in two nonevolved and three evolved populations (Fig. 2A). The mutation (3880A>C) locates within the putative RNA I of the origin of replication of pB1000 (36), a transcript that regulates the plasmid replication. This SNP was previously identified increasing the plasmid copy number (PCN) in wild-type heteroplasmid isolates of *Pasteurellaceae* (37). Indeed, the two nonevolved heteroplasmid populations exhibited the highest PCN (Fig. 2B), despite not being correlated with an increased fitness cost. These populations reduced their copies by half during the evolution without losing the heteroplasmid genotype (see Table S1 in the supplemental material). Additionally, in replicate III, the new allele appeared during the experiment reaching a final frequency of 9.28% without relevant modifications in its PCN.

10.1128/msphere.00184-22.4 TABLE S1 Plasmid copy number, phage copy number, and heteroplasmy. PCN, phage copy number and heteroplasmy genotype at the nucleotide 3880 of pB1000 (NC_019205.1) inferred by the coverage of their corresponding Illumina short read coverage. Download Table S1, XLSX file, 0.01 MB. Copyright © 2022 Ares-Arroyo et al. 2022 Ares-Arroyo et al. https://creativecommons.org/licenses/by/4.0/ This content is distributed under the terms of the Creative Commons Attribution 4.0 International license.

Some works have described PCN modifications during their compensation (15, 24). Hence, we characterized the overall PCN dynamics of pB1000 in our study. When pB1000 had been acquired recently, it showed 53.26 (SD, 35.71) copies per bacteria (Fig. 2B). Yet, at the end of the evolution, PCN decreased to 31.04 (SD, 11.56). Although no statistical difference was identified between the average PCN before and after the evolution [*t*_(4)_ = 1.83, *P* > 0.05], its variance showed a significant decrease [*F*_(4)_ = 7.63, *P* < 0.05], suggesting that PCN had been homogenized during the coadaptation. Our results indicate that the fitness cost of pB1000 was ameliorated during the experiment, regardless of its initial genotype or PCN. No additional mutations were identified in the replicon.

Recent works have proposed the transcriptional demand of MGEs as the source of its fitness cost and observed that transcriptomic modifications can play a major role in plasmid compensation (32). Therefore, we explored the transcriptome of pB1000. As already observed (38), plasmid-encoded genes were expressed significantly higher than the chromosome-encoded ones (Kolmogorov-Smirnov test, two-sided, *P* < 0.05) (Fig. 2C), which could be involved in its burden. However, the global transcriptional demand of pB1000 did not decrease during the evolution (Fig. 2C). Even more, after performing a differential expression analysis (see below), we observed that none of the plasmid-encoded genes show modifications in their expression after the plasmid adaptation to the host. Therefore, our results discarded any plasmid-mediated modification responsible for its compensation.

### Evolved populations accumulated few chromosome mutations.

**FIG 3:**
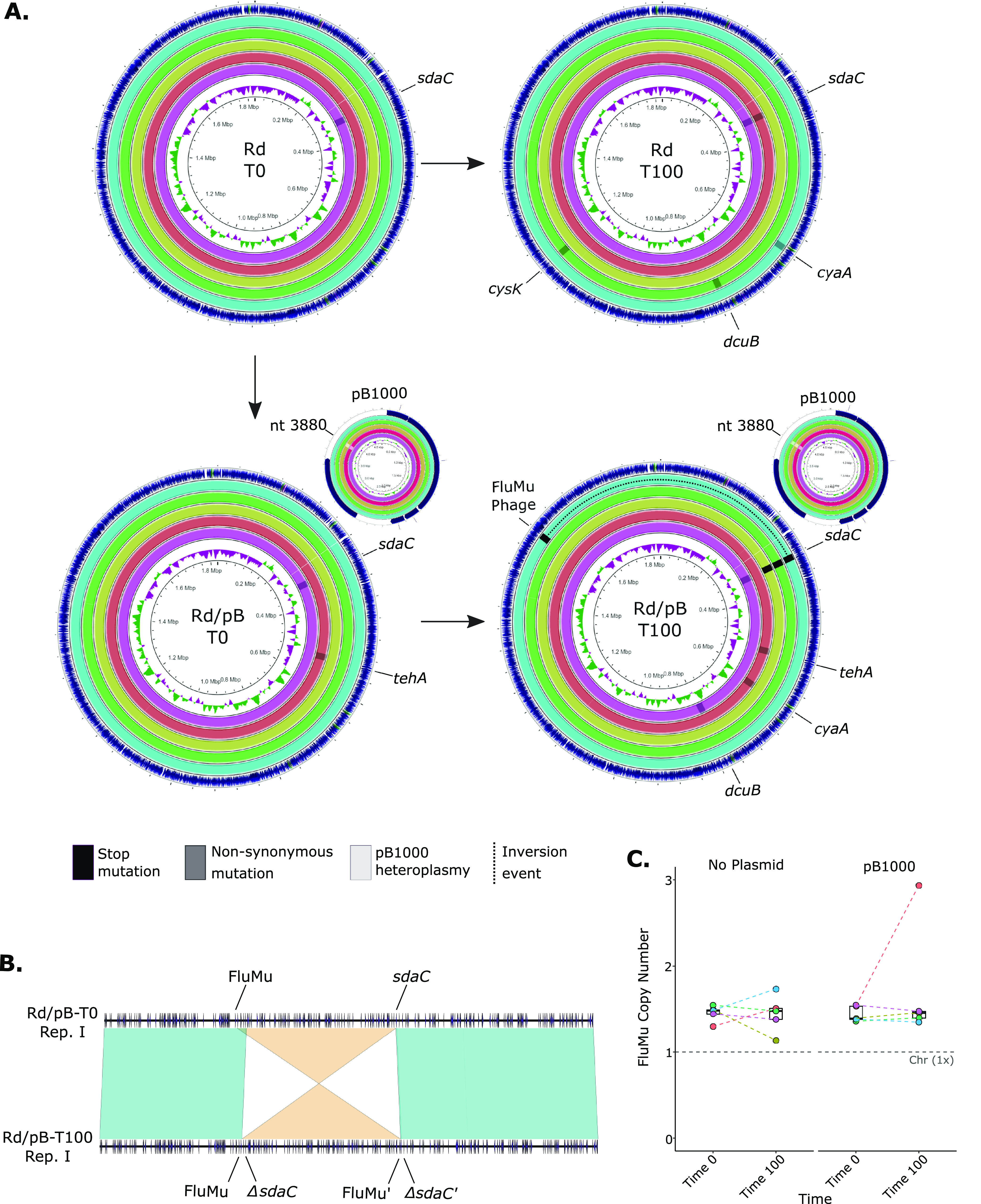
Genetic modifications among the Haemophilus influenzae chromosomes. (A) Chromosome mutations. Each circumference represents a chromosome, denoting its color the evolutionary replicate (outer circle, replicate I; inner circle, replicate V). The chromosomes are displayed in four groups, according to their evolutionary time point and plasmid status, and their locations within the figure are as follows: top left, plasmid-free and nonevolved bacteria (Rd-T0); top right, plasmid-free and evolved (Rd-T100); bottom left, plasmid-bearing and nonevolved (Rd/pB-T0); and bottom right, plasmid-bearing and evolved (Rd/pB-T100). The arrows between the groups represent the direction of experimental evolution. The plasmid pB1000 is represented as smaller circumferences next to the Rd/pB-T0 and Rd/pB-T100 chromosomes. Every mutation arising during the evolution is represented by a rectangle; the legend is at the bottom of the figure. The dashed black line represents an inversion event. The gene affected is indicated next to the circumference. (B) Inversion event within replicate I. The chromosome alignment of both nonevolved (Rd/pB-T0) and evolved (Rd/pB-T100) plasmid-bearing H. influenzae of replicate I. An inversion event is shown between the gene *sdaC* and the FluMu prophage, which is further detailed in the Supplemental Material. (C) Copy number of the FluMu prophage. Phage copy number of FluMu before (“Time 0” in *x* axis) and after (“Time 100” in *x* axis) the evolution. The colored dashed lines between points connect the copy number of the same evolutionary line. The gray horizontal dashed line represents the copy number of the chromosome (Chr.), estimated as 1.

As pB1000 showed neither genomic nor transcriptomic modifications, the host was suspected to be the only element responsible for the plasmid compensation. We looked for candidate compensatory mutations in the chromosome of H. influenzae, identifying 15 mutations in 12 populations, targeting 4 genes and an intergenic region (Fig. 3A; see Table S2 in the supplemental material). Only two mutations were described in nonevolved replicates, of which both were conserved along their following evolutions.

10.1128/msphere.00184-22.5 TABLE S2 Chromosome mutations in H. influenzae. Detailed information regarding the genomic modifications identified in the chromosome of Haemophilus influenzae RdKW20 over the experimental evolution. Download Table S2, XLSX file, 0.01 MB. Copyright © 2022 Ares-Arroyo et al. 2022 Ares-Arroyo et al. https://creativecommons.org/licenses/by/4.0/ This content is distributed under the terms of the Creative Commons Attribution 4.0 International license.

The gene that accumulated the largest number of mutations encoded a serine transporter (*sdaC*) in both plasmid-free and plasmid-bearing populations. This gene suffered stop mutations in three evolved pB1000-carrying bacteria, highlighting its disruption by an active lysogeny mediated by the prophage FluMu in one replicate (Fig. 3; see Fig. S1 and Text S1 in the supplemental material). However, *sdaC* presented nonsynonymous mutations in an evolved plasmid-free population and in every population originated from the Rd/T0 of replicate V. Moreover, the tellurite resistance gene *tehA* was mutated in an ancestral pB1000-bearing population and maintained during its evolution. Further mutations were identified in both plasmid-bearing and plasmid-free populations, possibly related to the aforementioned adaptation to the media after the initial evolution. These mutations targeted the genes encoding the adenylate cyclase (*cyaA*), the anaerobic C4-dicarboxylate transporter (*dcuB*), and 74 bp downstream of the cysteine synthase (*cysK*).

10.1128/msphere.00184-22.1 FIG S1 FluMu-mediated active lysogeny. At the top, shown is the chromosome alignment of both nonevolved (Rd/pB-T0) and evolved (Rd/pB-T100) plasmid-bearing H. influenzae of replicate I. An inversion event is shown between the points A and B of Rd/pB-T0 and C-D of Rd/pB-T100, which is further detailed in the four boxes at the bottom of the figure, with each one labeled with the respective letter (A, B, C, and D). Every arrow represents a gene, whereas the complete FluMu phage is represented as a blue block, annotated as “*36 Kb FluMu Phage*.” The number next to the arrows corresponds to the nucleotide number according to the reference sequence of Haemophilus influenzae RdKW20 (NC_000907.1). The darker green region represented in the gene *sdaC* (A, C, and D) corresponds to the 36-bp region repeated after the recombination event. Download FIG S1, PDF file, 0.04 MB. Copyright © 2022 Ares-Arroyo et al. 2022 Ares-Arroyo et al. https://creativecommons.org/licenses/by/4.0/ This content is distributed under the terms of the Creative Commons Attribution 4.0 International license.

10.1128/msphere.00184-22.9 TEXT S1 Active lysogeny. Detailed information on the active lysogeny mediated by the FluMu during the experimental evolution. Download Text S1, PDF file, 0.1 MB. Copyright © 2022 Ares-Arroyo et al. 2022 Ares-Arroyo et al. https://creativecommons.org/licenses/by/4.0/ This content is distributed under the terms of the Creative Commons Attribution 4.0 International license.

Hence, the only mutation associated with the plasmid-compensated bacteria was the truncation of *sdaC* (Table S2). This gene additionally exhibited nonsynonymous mutations in plasmid-free bacteria, suggesting that it could be further associated with the medium adaptation. Indeed, both Rd/pB-T0 and Rd-T0 show an increase in their relative fitness (although higher in the Rd/pB-T0), suggesting additional adaption to the media after the initial preadaptation. This finding suggests that, as recently observed in alternative models (31), mutations mediating niche adaptation could be additionally driving the plasmid compensation.

### The compensation of pB1000 entails transcriptomic modifications in the host.

After the genomic analysis, we focused on the transcriptomic modifications of the host. First, we explored the impact of the acquisition of pB1000 on the bacterial transcriptome through a differential expression (DE) analysis between the ancestral plasmid-free and the ancestral pB1000-bearing populations. Although 60 genes showed substantial variations (log_2_ fold change [LFC], >0.5), none of them achieved statistical significance (see Table S3 in the supplemental material). Notably, most of these genes are responsible for the general physiology of the cell, such as translation and metabolism, which have been already proposed as a source of plasmid-associated fitness cost (7, 8).

10.1128/msphere.00184-22.6 TABLE S3 Differential expression (DE) analysis. (A) DE analysis of the nonevolved populations. DE genes (DEGs) between the nonevolved plasmid-carrying populations (Rd/pB-T0) against the nonevolved plasmid-free populations (Rd-T0). (B) DE analysis of the evolved populations. DEGs between the evolved plasmid-carrying populations (Rd/pB-T100) against the evolved plasmid-free populations (Rd-T0). (C) DE analysis of the evolved and nonevolved plasmid-carrying populations. DEGs between the evolved plasmid-carrying populations (Rd/pB-T100) against the nonevolved plasmid-carrying populations (Rd/pB-T0). Download Table S3, XLSX file, 0.02 MB. Copyright © 2022 Ares-Arroyo et al. 2022 Ares-Arroyo et al. https://creativecommons.org/licenses/by/4.0/ This content is distributed under the terms of the Creative Commons Attribution 4.0 International license.

**FIG 4:**
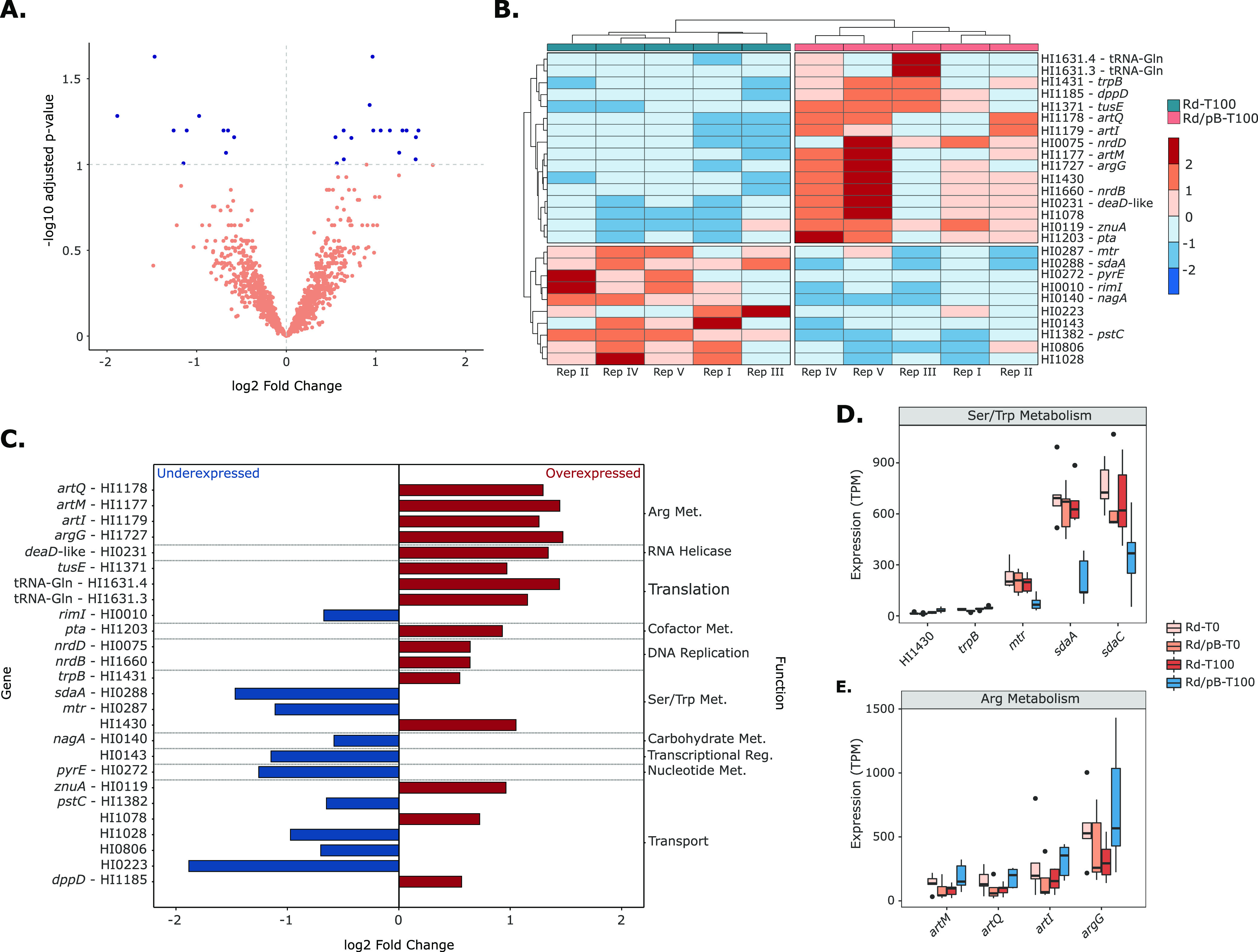
Differentially expressed (DE) genes after the compensation of pB1000. (A) DE genes of evolved populations. Volcano plot showing the DE genes between the evolved plasmid-bearing populations (Rd/pB-T100) against the evolved plasmid-free bacteria (Rd-T100). The *x* axis indicates the log_2_ fold change, while the *y* axis indicates the *P* value. Statistically significant genes (*P* < 0.1 and log2 fold change [FC], >0.5) are represented as blue dots, whereas the remaining genes are shown in orange. (B) DE genes after pB1000 compensation. Heatmap of the log2FC of the 26 DE genes in every replicate analyzed. The *x* axis represents the population and replicate, whereas the *y* axis represents the gene name. (C) Functional analysis of the DE genes after pB1000 compensation. Representation of the differentially expressed genes between bacteria evolved bearing pB1000 (Rd/pB-T100) and bacteria evolved without the plasmid (Rd-T100). Bars indicate the magnitude of differential expression (*x* axis); represented in red are the overexpressed genes and in blue are the underexpressed ones. On the left, the *y* axis indicates the gene name. On the right, the *y* axis indicates the function associated with the gene. (D) Expression of genes involved in Ser/Trp metabolism. Expression (TPM) of the genes responsible for the metabolism of serine and tryptophan at every time point of the evolution. (E) Expression of genes involved in Arg metabolism. Expression (TPM) of the genes responsible for the metabolism of arginine at every time point of the evolution. In every case, if the gene name was not available, the gene is described following the annotation of the reference genome of Haemophilus influenzae Rd KW20 (NC_000907.1).

Hence, we focused on the transcriptomic modifications involved in the plasmid compensation. Bacteria that had evolved carrying pB1000 showed 26 differentially expressed genes (DEGs) (10 under- and 16 overexpressed) compared with bacteria evolved without the plasmid (Fig. 4A and B; Table S3). Interestingly, 9 of the 26 genes were also DEGs compared with the ancestral pB1000-bearing populations (see Fig. S2 in the supplemental material; Table S3). Among the 26 genes, the most enriched process was the amino acid metabolism (Fig. 4C), which are mainly those pathways related to serine, tryptophan, and arginine (Fig. 4D and E). Furthermore, the expression of diverse transporters was also altered, despite most of them being poorly characterized. Among the remaining DEGs, it is worth mentioning the overexpression of two ribonucleotide reductases genes (*nrdD* and *nrdB*) due to their possible role in plasmid compensation. In summary, the analysis revealed that transcriptomic modifications affecting few chromosome-encoded genes occurred during the compensation of pB1000.

10.1128/msphere.00184-22.2 FIG S2 Differentially expressed (DE) genes after the compensation of pB1000. Volcano plot of the evolved plasmid-bearing populations (Rd/pB-T100) against nonevolved plasmid-carrying populations (Rd/pB-T0). The *x* axis indicates the log2 fold change, while the *y* axis indicates the *P* value. Statistically significant genes (*P* < 0.1 and log2FC > 0.5) are represented as blue dots, whereas the remaining genes are shown in orange. Download FIG S2, PDF file, 0.1 MB. Copyright © 2022 Ares-Arroyo et al. 2022 Ares-Arroyo et al. https://creativecommons.org/licenses/by/4.0/ This content is distributed under the terms of the Creative Commons Attribution 4.0 International license.

### H. influenzae exhibits specific metabolomic alterations after pB1000 compensation.

Due to the urgent need to understand the plasmid compensation phenomenon from an integrative multiomic approach (9, 38), we additionally performed a mass spectrometry-based multiplatform metabolomic study combining capillary electrophoresis coupled to mass spectrometry using a time-of-flight analyzer (CE-TOF/MS) and gas chromatography and liquid chromatography, coupled to mass spectrometry with a quadrupole-time-of-flight analyzer (GC-QTOF/MS and LC-QTOF/MS, respectively).

After comparing the abundance of the metabolites identified between the ancestral plasmid-free and ancestral plasmid-bearing populations, we observed no changes after pB1000 acquisition. In contrast, statistical analysis after pB1000 compensation showed a notable increase of serine within the evolved pB1000-carrying bacteria relative to both the ancestral (with and without pB1000) and evolved plasmid-free bacteria (one-way analysis of variance [ANOVA], Fisher *post hoc*, *P* < 0.05). This result was unveiled simultaneously by two analytical approaches (GC-QTOF/MS and CE-TOF/MS) (see Fig. S3 in the supplemental material), demonstrating that the serine abundance was actually altered during the plasmid compensation. The analysis of the remaining metabolites annotated through GC-QTOF/MS and CE-TOF/MS was determined as nonsignificant among sample groups.

10.1128/msphere.00184-22.3 FIG S3 Differential profile of the abundances of serine. Differential abundances of serine determined by GC-QTOF/MS (A) and CE-TOF/MS (B). The *x* axis indicates the population, whereas the *y* axis indicates the normalized abundance. Statistical significance of the serine abundances in population Rd/pB-T100 was verified through one-way parametric ANOVA with LSD Fisher *post hoc* analysis (*P* < 0.05). Download FIG S3, PDF file, 0.04 MB. Copyright © 2022 Ares-Arroyo et al. 2022 Ares-Arroyo et al. https://creativecommons.org/licenses/by/4.0/ This content is distributed under the terms of the Creative Commons Attribution 4.0 International license.

**FIG 5:**
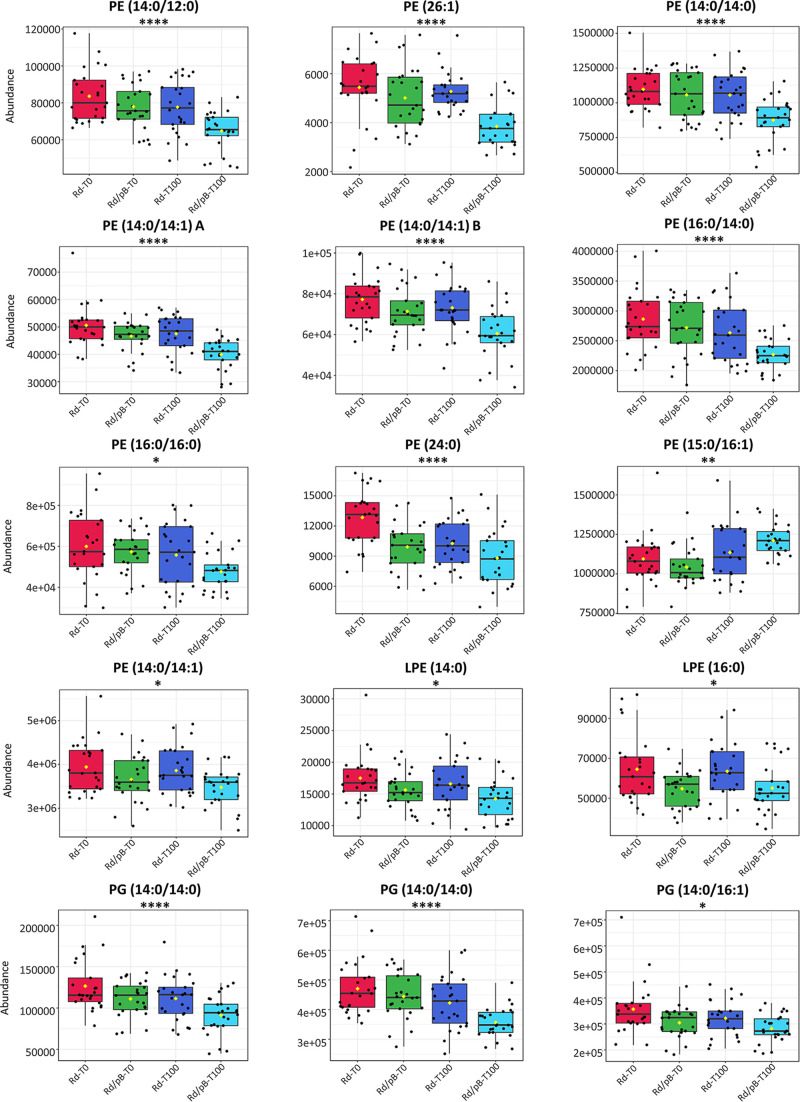
Differential profile of the abundances of statistically significant phospholipids. Statistically significant phospholipids were annotated as phosphatidyletanolamines (PEs), lysophosphatidylethanolamines (LPEs) and phosphatidylglycerols (PGs); the specific compound is represented at the top of each figure. The *x* axis indicates the population, whereas the *y* axis indicates the abundance. The statistical significance observed in the evolved plasmid-bearing populations (Rd/pB-T100) is indicated through the asterisks, as follows: *, *P* < 0.05; **, *P* < 0.01; ***, *P* < 0.001; ****, *P* < 0.0001; the *P* value is FDR adjusted.

Interestingly, a comparative analysis of the lipidome revealed decreased abundances of specific phospholipids (phosphatidylglycerols, phosphatidylethanolamines, and lysophosphatidylethanolamines) within the evolved pB1000-carrying bacteria (Fig. 5). The most significantly altered phospholipids possessed a C_14:0_ saturated fatty acyl moiety, and when a determination of the fatty acyl chains was not possible, lower-level significant annotations were compatible with the presence of the C_14:0_ fatty acyl moiety. These results exhibit further modifications during plasmid compensation related to myristic acid, whose origin lies beyond genomic and transcriptomic modifications.

## DISCUSSION

Understanding the molecular mechanisms of coadaptation between plasmids and their host is a current priority in microbiology, which is crucial to fight antimicrobial-resistant bacteria. With this purpose, we combined experimental evolution with a multiomic analysis using Haemophilus influenzae and pB1000 as a model. Interestingly, when we attempted to understand the source of pB1000-associated fitness cost, none of the tendencies identified, neither transcriptomic nor metabolomic, were statistically significant. Indeed, it was shown recently that the acquisition of multidrug resistance plasmids is not always associated with severe transcriptomic modifications (39), and the role of Haemophilus spp. as an ancient host of ColE1-like plasmids might justify its limited burden (36). Actually, pB1000 is spread among different genera of *Pasteurellales* (34, 35, 37), is 100% stable over 300 generations (37), and exhibits a remarkable GC percentage to its host (40).

However, despite not identifying the source of the cost of pB1000, we found that it did produce a 5% fitness cost compensated in ~100 generations. Previous models have shown that plasmid-host coadaptation might involve plasmid-mediated modifications (18, 19, 24, 30), but our integrative analysis showed that there were no compensatory modifications in the plasmid. These results are consistent with an early work aiming to comprehend the compensation of the ColE1-like plasmid pACYC184 in Escherichia coli (12). Multicopy plasmids hinder the fixation of new alleles within the cell (41). Thus, host modifications are more suitable to quickly ameliorate the fitness cost of the multicopy plasmid. Furthermore, this information explains the increased permissiveness exhibited to additional ColE1-like replicons after compensating pB1000 and facilitating the appearance of multiresistance (17).

In contrast to already published models (18, 23), our integrative analysis concluded that the compensatory evolution of pB1000 involved limited modifications in the host. Among all the modifications, amino acid metabolism was the main common denominator implicated in the plasmid adaptation, mainly the serine, tryptophan, and arginine pathways. This finding was particularly exciting, as Chant and Summers (42) already demonstrated that the genesis of ColE1-like plasmid dimers rises the concentration of indole within the cell by increasing the affinity of the tryptophanase to its reactant. The intracellular accumulation of indole acts as a cell cycle regulator, inhibiting the cell growth and providing time for the activity of the dimer resolution machinery, thus avoiding the phenomenon known as the dimer catastrophe despite hindering the fitness of the host (43).

**FIG 6:**
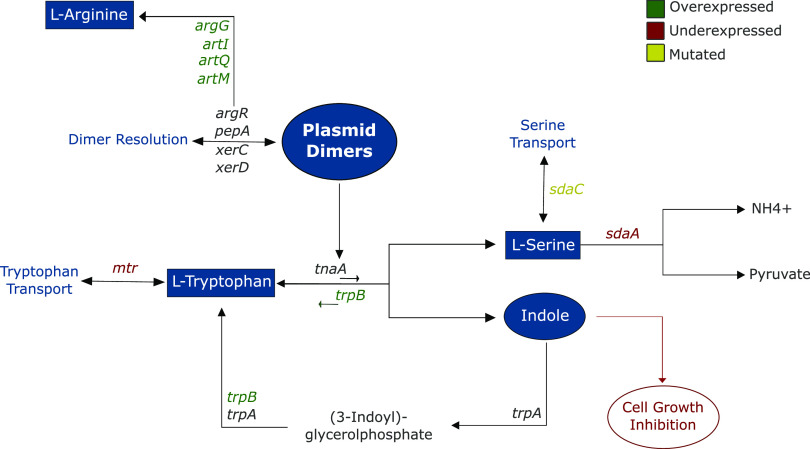
Modifications within the amino acid metabolism. Schematic representation of the pathways of l-serine, l-tryptophan, and l-arginine involved in pB1000 compensation and their connection to plasmid dimer formation and resolution. The arrows connecting metabolites indicate the direction of enzymatic reactions. The genes involved in the reactions are shown next to the arrows. The font color of the gene names indicates their molecular modifications after the plasmid compensation, as follows: green, overexpressed; red, underexpressed; yellow, mutated; and black, nonmodified.

Our transcriptomic analysis revealed that compensated pB1000-carrying bacteria showed an overexpression of the tryptophan synthase (*trpB*), whose function counteracts the function of the tryptophanase, implying that plasmid-compensated bacteria would decrease the intracellular concentration of serine and indole (Fig. 6). However, these populations show increased levels of serine, surely due to the following additional modifications within this pathway, which may compensate for the unbalanced amino acid pool: underexpression of *sdaA*, responsible for serine catabolism; overexpression of the tryptophan transporter *mtr*; and diverse stop mutations in the serine extracellular transporter *sdaC*. Therefore, our results strongly suggest that the compensatory evolution of ColE1-like plasmids occurred counteracting the cell cycle repression produced by plasmid dimers within the cell. Remarkably, one of the aforementioned mutations was triggered by the replication of the FluMu phage, which exposes the interaction between different MGEs as a common strategy for plasmid amelioration (23).

This host-mediated plasmid adaption would be prejudicial to the replicon, as dimers are unstable within populations. However, our results indicate that pB1000 has been 100% stable, and furthermore, it exhibits a more homogeneous PCN among the evolved bacteria, which is hardly understandable without an efficient dimer resolution system. Our hypothesis is that a shorter cell cycle and its subsequent shorter time window to resolve dimers might have occurred in parallel to a more efficient resolution system. This higher efficiency related to the arginine repressor ArgR, which is physically needed for the ColE1 dimer resolution together with the recombinases XerC and XerD (43). In our work, genes negatively regulated by ArgR (*artQ*, *artM*, *artI*, and *argG*) showed underexpression tendencies during pB1000 acquisition (Table S3), and were significantly overexpressed after its compensation (Fig. 4C; Table S3). These findings suggest that ArgR might be sequestered in its dimer resolution function, rather than in the arginine biosynthesis pathway.

Nevertheless, additional modifications were identified with candidate roles in the plasmid compensation, highlighting the ribonucleotide reductases (RNRs; *nrdA*, *nrdB*, and *nrdD*). RNRs are extremely regulated enzymes, which are expressed only when the initiation mass of the cell triggers the chromosome replication (44). It has been shown that unregulated RNRs or unbalanced deoxynucleoside triphosphate (dNTP) pools hamper DNA replication and cell division (45). As ColE1-like plasmids increase the dNTP demand and their replication is not synchronized with the chromosome (46), their maintenance may produce such an imbalance. Thus, our results suggest that an overexpression of the RNRs could be involved in the compensation of pB1000 as well. Indeed, RNRs have been described in various MGEs, such as plasmids (47) and phages (48), suggesting that they are an extended mechanism of amelioration for MGEs. The relationship of the remaining modifications with the plasmid/host coadaptation is more intriguing, as most of the genes are still poorly characterized.

Lastly, our analysis showed that not only genomic and transcriptomic modifications occurred but also a rearrangement of the membrane phospholipids arose during the evolution. The results suggest that an underlying biological process involving the myristic acid is selectively regulated upon the coevolution of H. influenzae with pB1000. Notably, lipopolysaccharide (LPS) modifications have been associated with a decreased susceptibility to phage predation (49), and myristoyl substitutions in the lipid A of H. influenzae have been described as a major lipid A residue (50), which is critical factor involved in the endotoxic activity of Gram-negative bacteria (51). This finding implies that further nongenomic and nontranscriptomic modifications mediate the plasmid-host coadaptation, whose consequences could even extend to the phage resistance and virulence of the bacteria.

Our results provide new insights into the adaptation of ColE1 plasmids to their hosts, bringing together diverse works on this family of replicons (e.g., the rapid cost amelioration [35], a host-mediated compensation [12, 17], or the role of ColE1 in the amino acid metabolism [42, 43]). Yet, our findings suggest that the mechanism involved in the ColE1 plasmid compensation might be specific to this family of plasmids since it responds to a dimer resolution system identified only in these multicopy replicons. This suggestion is in line with the fact that each plasmid-host pairing incurs typically in the modification of specific genes, despite affecting the same global functions (52); while some plasmid/host adaptive modifications in the literature enhance the horizontal transfer of the plasmid, other modifications (such as those identified in the present work, including amino acid and nucleotide metabolism or several transporters) likely benefit the vertical transmission of the plasmid (52). This idea is reasonable since ColE1 plasmids cannot autonomously conjugate and, hence, would benefit from an increased vertical transmission within the population.

In conclusion, our work showed that minimal modifications in the host occurred during the coevolution of H. influenzae and pB1000. Its compensation was driven by diverse mechanisms, such as counteracting the plasmid-dimer effects to the cell growth, modifying the RNR expression, and amending the phospholipids within the membrane. The combination of all the modifications, mainly transcriptomic and metabolomic, was ultimately responsible for the plasmid amelioration. This work unveils the need to study the compensatory evolution from an integrative perspective if we aim to completely understand the molecular mechanisms involved in the plasmid-host coadaptation.

## MATERIALS AND METHODS

### Bacterial strains and culture conditions.

In our model, we used the bacterial strain Haemophilus influenzae RdKW20 (GenBank accession no. NC_000907.1) (33) and the ColE1-like plasmid pB1000 (NC_019205.1) (34). Bacteria were cultured on chocolate agar PolyViteX plates (bioMérieux, France) and in Haemophilus test medium (HTM) broth (Francisco Soria Melguizo, S.A., Spain) at 37°C under microaerophilic conditions (5% CO_2_) with continuous shaking at 125 rpm. H. influenzae was transformed by electroporation using an Eporator device (Eppendorf AG, Hamburg, Germany) with the following conditions: 2.5 kV/cm, 25 μF, and 200 Ω. Transformants were selected by supplementing the media with 64 mg/L of ampicillin (Sigma-Aldrich, St. Louis, MO).

### Experimental design.

We designed an experimental evolution performed in five parallel replicates. The experimental evolution procedure was maintained throughout the whole experiment, diluting the grown cultures 1:1,000 every 24 h into 2 mL of fresh medium under the aforementioned conditions. First, we evolved the populations of plasmid-free H. influenzae RdKW20 (“*Rd-Pre*”) in HTM broth for 10 days (~100 generations). The resulting adapted populations (“*Rd-T0*”) were transformed with pB1000, obtaining the populations “*Rd/pB-T0*,” which should show the fitness cost imposed by the acquisition of the plasmid. Finally, Rd/pB-T0 were evolved in the absence of ampicillin for 100 generations (“*Rd/pB-T100*”). In addition, Rd-T0 was similarly evolved for 100 generations without the plasmid (“*Rd-T100*”), so we could include as a control plasmid-free bacteria that had evolved the same generations as the plasmid-carrying bacteria.

In summary, the populations included in the integrative multiomic analysis were Rd-T0 (plasmid free; nonevolved), Rd/pB-T0 (pB1000; nonevolved), Rd/pB-T100 (pB1000; evolved), and Rd-T100 (plasmid-free; evolved). The Rd-Pre populations were also used for the fitness determination.

### Fitness determination.

The relative fitness of the populations was determined by competition experiments between plasmid-free and plasmid-carrying bacteria at different time points of the experimental evolution. First, we analyzed the initial adaptation to the media competing both Rd-Pre and Rd-T0 against a previously described pB1000-carrying H. influenzae RdKW20 (35). For the determination of the fitness cost of pB1000 and its compensatory evolution, Rd/pB-T0 and Rd/pB-T100 were competed against their ancestral Rd-T0. Furthermore, Rd/pB-T100 was also competed against their respective Rd-T100.

Every competition assay was performed as described previously (35), mixing 10^6^ CFU of each bacterium in 2 mL of HTM and letting the culture grow for 24 h at 37°C, 125 rpm, and 5% CO_2_. The competitions lasted for 5 days, in which every 24 h a 1:1,000 dilution was performed into new fresh media. Samples were taken at time zero and every 24 h and plated onto chocolate agar. Then, the proportion of resistant colonies was calculated by replica plating of 80 to 100 colonies on chocolate agar plates supplemented with 64 mg/L of ampicillin. The selection coefficient (*s*) was calculated as the slope of the linear regression model *s* =* ln(CI)/t*, where *t* is the time measured in bacterial generations calculated as the log_2_ of the dilution factor; and *CI* is the competition index, calculated every day as the ratio between the resistant and susceptible strains at *t_1_*, divided by the same ratio at *t_0_*. The relative fitness (*w*) was then calculated with the formula *w *= *1* + *s*. The selection coefficient *s* was calculated as the mean from three independent replicates of the competition assays. For data visualization (Fig. 1), the results were standardized taking as reference their corresponding Rd-T0 in each of the evolved lines.

### Genomic DNA extraction.

The genomic DNA extraction for the whole-genome sequencing was performed using the Wizard genomic DNA purification kit (Promega Corp., Madison, WI) following the “Isolation Genomic DNA from Gram Negative Bacteria Protocol.” DNA concentration was measured by a QUBIT instrument (Invitrogen Corp., Carlsbad, CA), and DNA quality was assessed by a Nanodrop instrument (Thermo Fisher Inc., Waltham, MA), following the manufacturer’s instructions.

### Whole-genome sequencing by Illumina and data processing.

Illumina sequencing of Rd-T0, Rd/pB-T0, Rd/pB-T100, and Rd-T100 from the five evolved lines was performed in the Denmark Technical University (DTU; Lyngby, Denmark). The library preparation used was Nextera XT (Illumina Inc., San Diego, CA), and the sequencing was performed in a MiSeq platform (Illumina Inc.), generating 2 × 250-bp paired reads (see Table S4 in the supplemental material). The read quality was assessed with FastQC, version 0.11.3 (53), and low-quality ends were trimmed using Trimmomatic, version 0.33 (54). The Illumina short reads generated were used to perform a SNP calling analysis using as reference the genome of Haemophilus influenzae RdKW20 (NC_000907.1). The reads of every sample were mapped to the indexed reference using Bowtie 2, version 2.3.4.1 (55), and the SNP calling analysis was performed with SAMtools, version 1.7 (56). Each evolved replicate was analyzed separately. All the candidate mutations were tested afterward by PCR and sequenced via Sanger technology in Macrogen (Macrogen Spain Inc., Spain). Additionally, we used the short read coverage to infer the copy number from both the plasmid pB1000 and the H. influenzae RdKW20 prophages ϕflu and FluMu (57) (Table S1).

10.1128/msphere.00184-22.7 TABLE S4 Data of the whole-genome sequencing (WGS) analysis. (A) Data of the short reads generated by Illumina WGS. (B) Data of the long reads generated by Nanopore WGS. (C) Data of the hybrid assembly combining the Illumina short reads and Nanopore long reads. Download Table S4, XLSX file, 0.01 MB. Copyright © 2022 Ares-Arroyo et al. 2022 Ares-Arroyo et al. https://creativecommons.org/licenses/by/4.0/ This content is distributed under the terms of the Creative Commons Attribution 4.0 International license.

### Whole-genome sequencing by Nanopore and data processing.

Samples were additionally sequenced using a MinION device (Oxford Nanopore Technologies Ltd., Oxford, UK). Genomic libraries were performed using the EXP-NBD103 and SQK-LSK108 kits (Oxford Nanopore Technologies Ltd.) following the 1D Native barcoding genomic DNA protocol with a FLO-MIN106 flow cell. Sequenced reads were basecalled with the MinKNOW software (Oxford Nanopore Technologies Ltd.), and the demultiplexing process was performed with the Fastq Barcoding workflow of the Epi2Me interface (Metrichor Ltd., Oxford, UK). The adaptors and barcodes were trimmed with the software Porechop version 0.2.3 (58). The Nanopore reads were used together with the Illumina reads to perform a hybrid assembly using the software Unicycler, version 0.4.0 (59), closing the complete genome sequences with Bandage, version 0.8.1 (60) (Table S4). Chromosome mutations among the samples were identified by multiple genome alignments performed with the progressive Mauve algorithm (61) and visualized with the Geneious Benchmark, version 2019.0.4. Mutations were annotated through homology with the already annotated Haemophilus influenzae RdKW20 genome (NC_000907.1). Like in the prior SNP calling analysis, all the candidate mutations were validated afterward by PCR and sequenced via Sanger in Macrogen (Macrogen Spain, Madrid, Inc., Spain).

### RNA extraction.

RNA extractions were obtained from the five replicate populations of Rd-T0, Rd/pB-T0, Rd/pB-T100, and Rd-T100. Bacteria were cultured in HTM media and grown overnight at 37°C, 125 rpm, and 5% CO_2_. Overnight cultures were diluted 1:100 in fresh HTM and incubated until they reached an optical density at 600 nm (OD_600_) of 0.6 and mid-late exponential phase of the growth curve. Then, we added RNAprotect bacterial reagent (Qiagen, Hilden, Germany) to the cultures according to the manufacturer’s instructions and proceeded to the RNA extraction with the RNeasy minikit (Qiagen). Genomic DNA contamination was tested with both the QUBIT instrument (Invitrogen Corp., Carlsbad, CA) and by performing PCRs of the H. influenzae chromosomal gene *rpoB* (Gene identifier [ID] 56844421). Additional on-column DNase I digest treatments (Qiagen) were performed after the extractions until the samples were completely DNA-free. The RNA concentration of the samples was determined by a QUBIT instrument (Invitrogen Corp.), and its quality was determined by Nanodrop (Thermo Fisher Inc., Waltham, MA) and gel electrophoresis.

### RNA-Seq analysis.

The rRNA depletion and subsequent transcriptome sequencing (RNA-Seq) library and sequencing was carried out by Vertis (Vertis Biotechnologie AG, Freising, Germany). The library preparation was performed with TruSeq and the sequencing platform employed was Illumina NextSeq 500, generating 75-bp-long single reads (see Table S5 in the supplemental material). The data processing consisted of a first step of quality control with the software FastQC, version 0.11.3 (53). Then, the reads generated were aligned using the mapper Bowtie 2 version 2.3.4.1 (55) to the indexed reference genome NC_000907.1 for Haemophilus influenzae RdKW20 and NC_019205.1 for the plasmid pB1000. The quality control of the alignments and the gene counts were assessed with HTSeq (62). The differential expression analysis was performed in R, version 3.6.1, with the R package DESeq2, version 1.26.0 (63).

10.1128/msphere.00184-22.8 TABLE S5 Data of the RNA-Seq reads. Data of reads generated in the RNA-Seq analysis. Download Table S5, XLSX file, 0.01 MB. Copyright © 2022 Ares-Arroyo et al. 2022 Ares-Arroyo et al. https://creativecommons.org/licenses/by/4.0/ This content is distributed under the terms of the Creative Commons Attribution 4.0 International license.

### Metabolite extraction.

Samples were obtained from five biological replicates from each evolution line and time point. Bacteria were cultured overnight in 2 mL of HTM at 37°C, 125 rpm, and 5% CO_2_. The bacteria were diluted 1:100 into fresh HTM and cultured until they reached an OD_600_ of 0.6, at the mid-late exponential phase of the growth curve. Samples were centrifuged (1,080 × *g* for 3 min at room temperature [RT]) and the supernatant discarded. The bacterial pellet was resuspended in 800 μL of phosphate-buffered saline (PBS) and centrifuged (1,080 × *g* for 3 min at RT), and the supernatant was discarded. Lastly, 200 μL of cold methanol (−20°C) was added to the bacteria for quenching and maintained at −80°C until the extraction of metabolites that was carried out in the Centre for Metabolomics and Bioanalysis (CEMBIO; San Pablo CEU University, Madrid, Spain).

To enhance the coverage, a double extraction was performed. Briefly, 17.2 μM *p*-Cl-phenylalanine (Sigma-Aldrich, Steinheim, Germany) in 140 μL Milli-Q water was added to the pellets conserved in methanol. Samples were lysed in a UP 200S ultrasonicator equipped with an S2 probe (Dr Hielscher GmbH, Stahsdorf, Germany) with freeze-thaw cycles for a polar metabolite extract (H_2_O:MeOH 1:1.43, vol/vol). Samples were placed in the ultrasonicator probe and placed directly in liquid N_2_ (50 s) after 20 pulses of 0.5 s (amplitude, 60%). This process was repeated 20 times. Lysed samples were kept on ice (15 min). Subsequently, samples were centrifuged to precipitate all possible debris (12,600 × *g* for 30 min at 4°C), and the polar supernatant was collected for further GC-EI-QTOF/MS and CE-ESI(+)-TOF/MS analyses. The remaining debris pellet was stored at −80°C for a second, nonpolar extraction. Debris pellets were thawed under cold ice, mixed with 200 μL of MeOH:methyl *tert*-butyl ether (MTBE; 1:1, vol/vol), and resuspended by three pulses of an ultrasonicator probe (amplitude, 20; cycle, 0.5). Debris samples were briefly placed on ice (1 min) and subsequently vortexed for 30 min; afterward, samples were left at RT for metabolite extraction (5 min). Then, they were centrifuged (12,600 × *g* for 30 min at RT) and 100 μL of each nonpolar metabolite extract was transferred to liquid chromatography-mass spectrometry (LC/MS) vials, which were quickly capped for LC-ESI-QTOF/MS lipidomics analysis.

### CE-TOF/MS analysis and data processing.

Prior to sample analysis, 120 μL of each H_2_O:MeOH extract (1:1.43, vol/vol) was evaporated to dryness using a vacuum concentrator. The dried samples were resuspended in 60 μL of Milli-Q water containing 0.1 mM formic acid (Sigma-Aldrich, Steinheim, Germany) and 0.2 mM methionine sulfone (Sigma-Aldrich) by vortexing for 1 min. After subsequent centrifugation (12,600 × *g* for 15 min at 4°C), the solution was transferred to CE/MS glass vials which were centrifuged again (4,000 × *g* for 20 min at 4°C) to precipitate any possible suspended solid particle. Samples were analyzed by CE-ESI(+)-TOF/MS using a CE system (Agilent 7100) coupled to a TOF/MS system (Agilent 6224), following a methodology previously described (64) (see Text S2 in the supplemental material). CE-TOF/MS data processing consisted of compound deconvolution, annotation of metabolites using an in-house library (65) and online databases (66), blank subtraction, internal standard normalization, and data filtering. (Text S2).

10.1128/msphere.00184-22.10 TEXT S2 Metabolomic analysis. Detailed information regarding the materials and methods of the metabolomic analysis. Download Text S2, PDF file, 0.1 MB. Copyright © 2022 Ares-Arroyo et al. 2022 Ares-Arroyo et al. https://creativecommons.org/licenses/by/4.0/ This content is distributed under the terms of the Creative Commons Attribution 4.0 International license.

### LC-QTOF/MS analysis and data processing.

MeOH:MTBE extracts were analyzed in a high-performance liquid chromatography (HPLC) system equipped with a degasser, two binary pumps, and a thermostated autosampler coupled to a 6545 QTOF/MS system (Agilent Technologies, CA), following an adapted version of the Lipid Annotator method described by Agilent Technologies (67) (Text S2). LC-QTOF/MS data processing consisted of deconvolution of full-scan MS data from both ESI+ and ESI− polarities, mapping of MS/MS annotations to potential compounds detected in full-scan MS data, manual completion of lipid profiles of the distinct lipid classes identified by MS/MS analysis using online databases (66), targeted compound integration, blank subtraction, and data filtering (Text S2).

### GC-QTOF/MS analysis and data processing.

A total of 100 μL of each MeOH:H_2_O polar extract (1:1.43, vol/vol) was evaporated to dryness under high vacuum. The obtained dried extracts were derivatized as described previously (68). Briefly, aldehyde and keto groups were first converted to *O*-methyloximes by reaction with 10 μL pyridine containing 15 mg/mL i-methoxyamine (Sigma-Aldrich, Steinheim, Germany) for 16 h at RT. Then, acid hydrogen-containing metabolites were trimethylsilylated by reaction with 10 μL *N*,*O*-bis(trimethylsilyl)trifluoroacetamide-trimethylchlorosilane 99:1 (Sigma-Aldrich) to enhance the GC/MS metabolite coverage. Samples were incubated for 60 min at 70°C and cooled down at RT for 30 min. Subsequently, samples were reconstituted in 50 μL of 71.8 μM tricosane in *n*-heptane for GC/MS analysis. Sample analysis was performed on an Agilent Technologies 7890B GC system coupled to an Agilent Technologies 7250 accurate mass Q/TOF analyzer equipped with an electron ionization (EI) source. Sample analysis was adapted from a methodology described previously (68) (Text S2). GC-QTOF/MS data processing consisted of feature deconvolution, metabolite annotation using in-house and commercially available spectral libraries (69, 70), compound integration of annotated metabolites, blank subtraction, internal standard normalization, and data filtering (Text S2).

### Metabolomic statistical analysis.

One-way, parametric ANOVA with false discovery rate (FDR) multiple testing correction and least significant difference (LSD) Fisher *post hoc* analysis was used to evaluate statistically significant differences among the populations. The normal distribution and homoscedasticity of variances were checked by Kolmogorov-Smirnov and Levene tests, respectively. Individual statistical tests were performed for the distinct data matrices obtained from GC-EI-QTOF/MS, CE-ESI(+)-TOF/MS, LC-ESI(+)-QTOF/MS, and LC-ESI(−)-QTOF/MS analyses.

### Data availability.

Raw Illumina short reads, raw Nanopore long reads, and raw RNA-Seq reads were deposited in the European Nucleotide Archive (ENA) under the project PRJEB44283. The individual accession no. of each sample is available in the Table S4.
