# Supplementary figures and images for: Genomics, Transcriptomics, and Metabolomics Reveal That Minimal Modifications in the Host Are Crucial for the Compensatory Evolution of ColE1-Like Plasmids
Source: mSphere. 2022 Nov 23;7(6):e00184-22. doi: 10.1128/msphere.00184-22 (PMC9769657; doi:10.1128/msphere.00184-22)

# Replicate I: Rd/pB-T0 and Rd/pB-T100

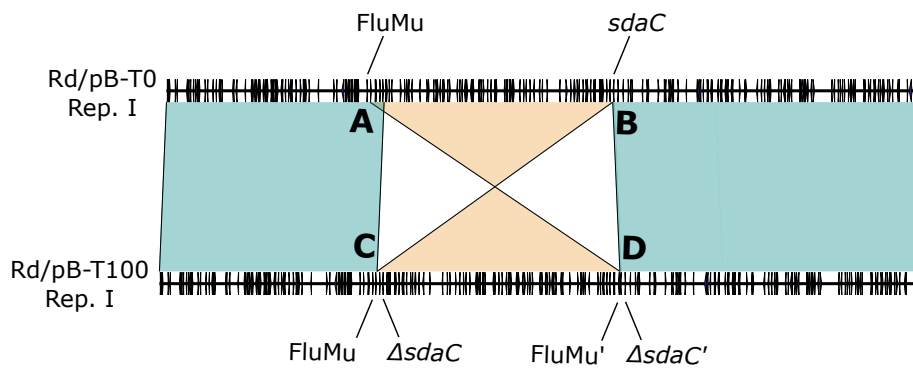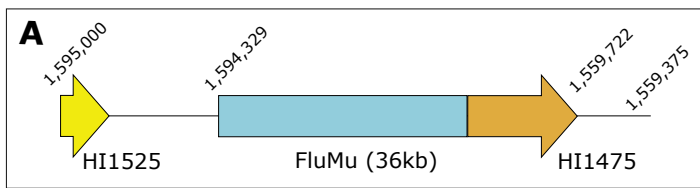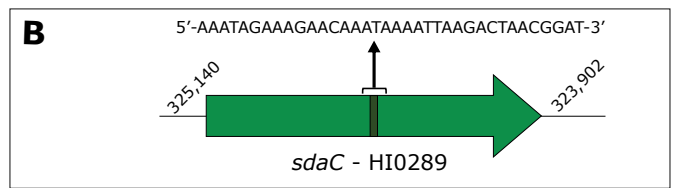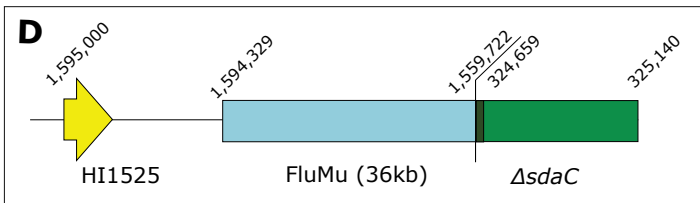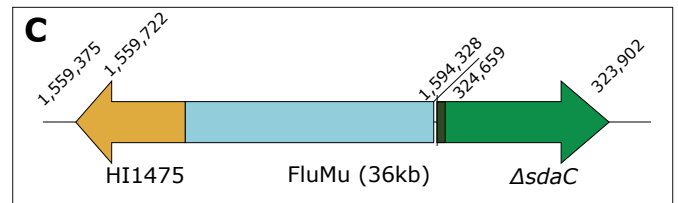

Supplement: FIG S1 [file msphere.00184-22-s0003.pdf]

**A.**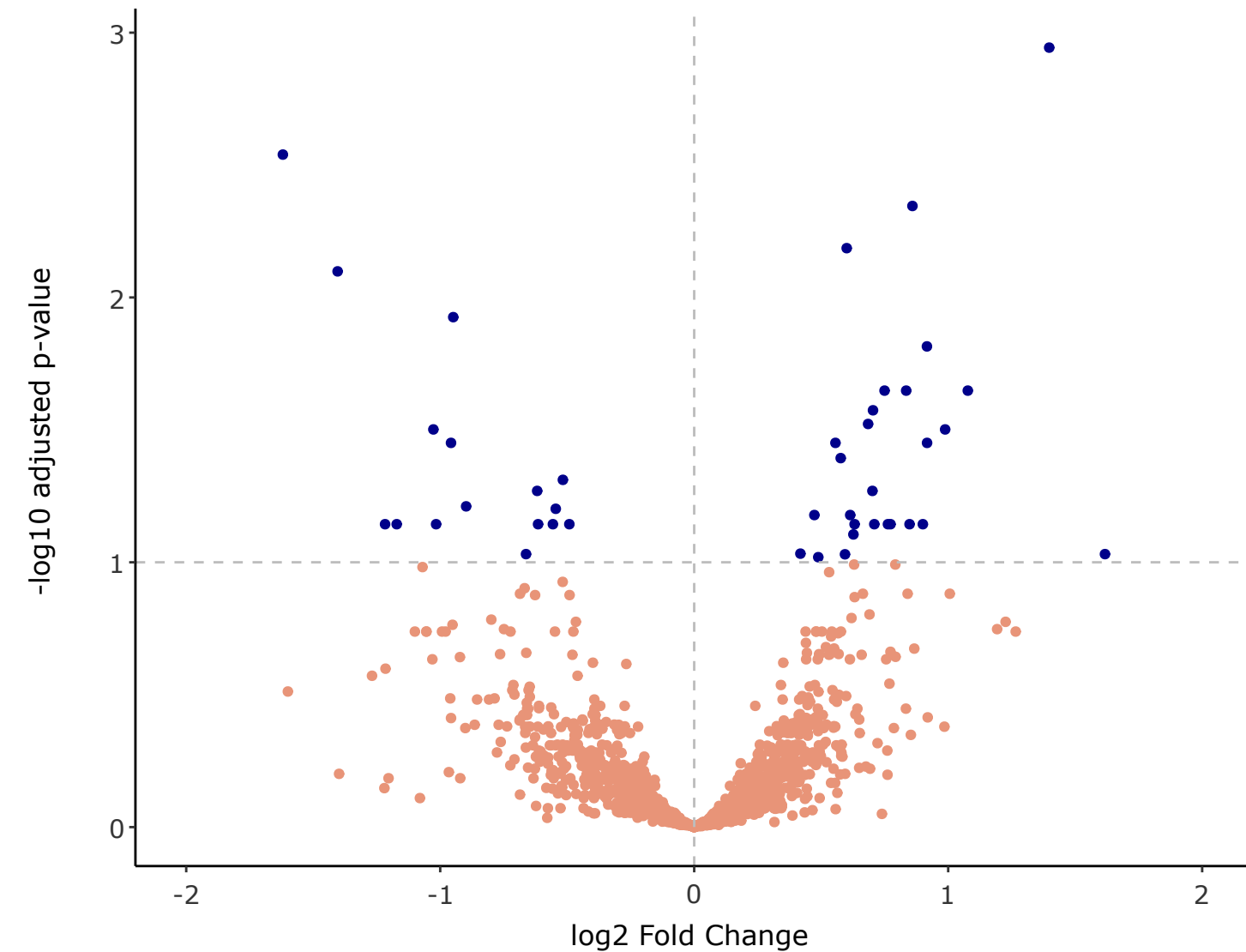**B.**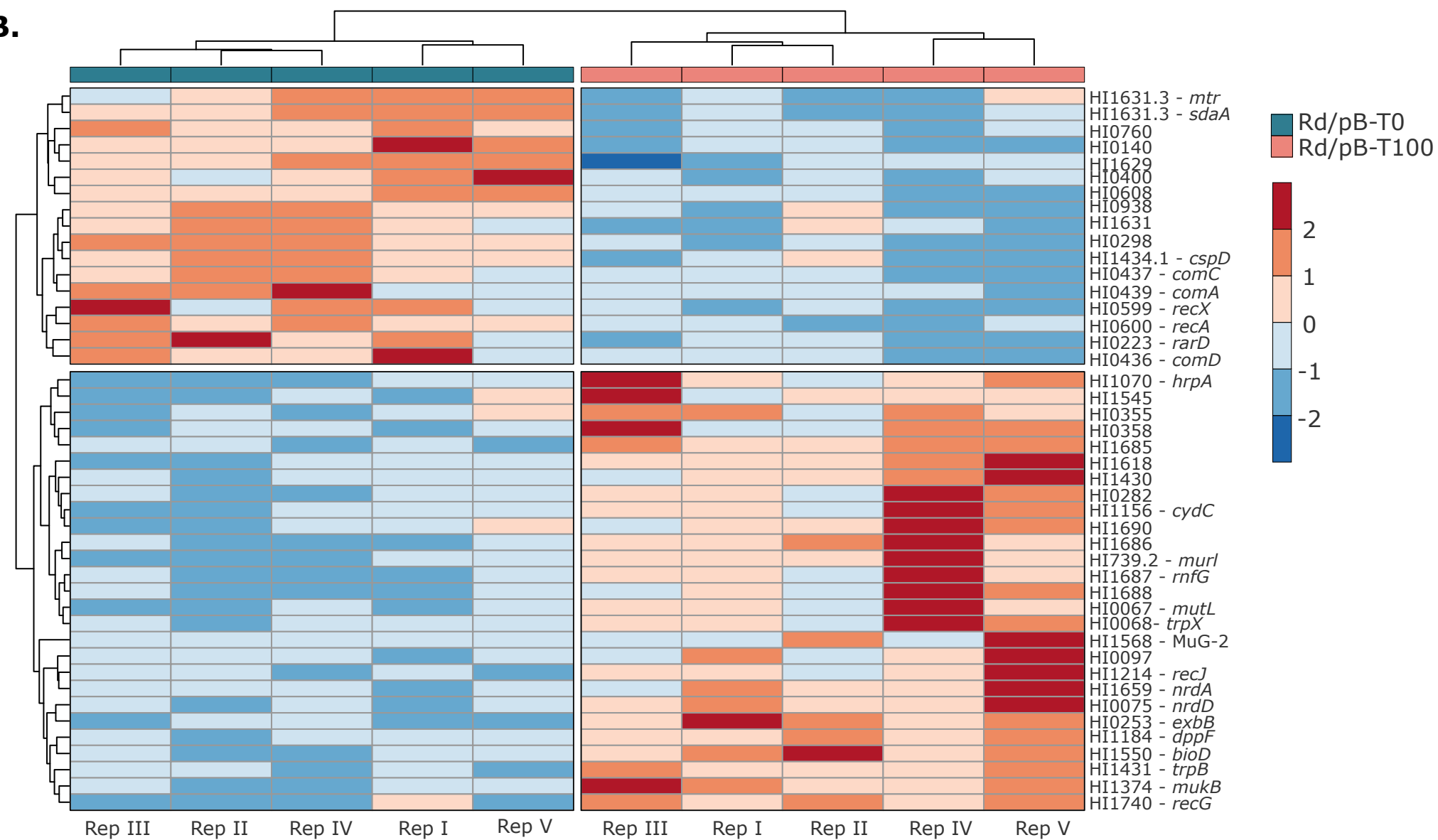

Supplement: FIG S2 [file msphere.00184-22-s0004.pdf]

**A.**

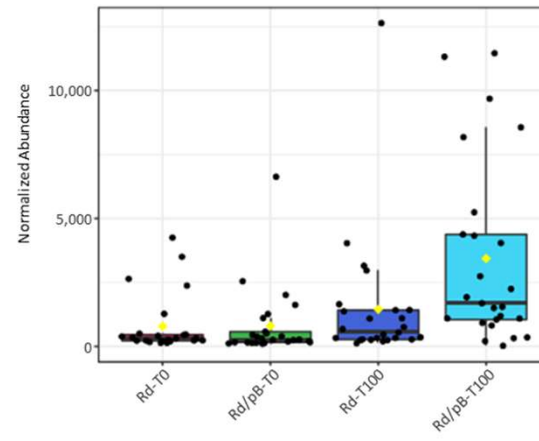

**B.**

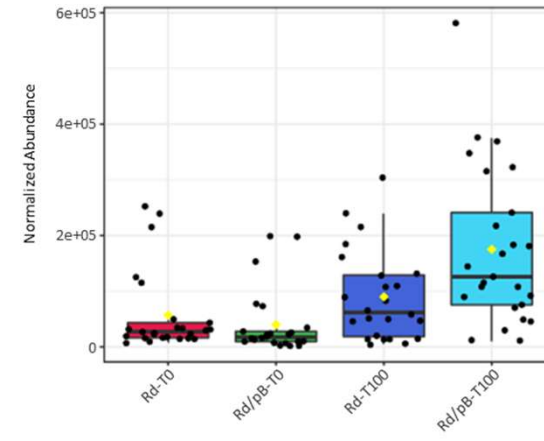

Supplement: FIG S3 [file msphere.00184-22-s0005.pdf]
