# Supplementary material for: Genomics, Transcriptomics, and Metabolomics Reveal That Minimal Modifications in the Host Are Crucial for the Compensatory Evolution of ColE1-Like Plasmids
Source: mSphere. 2022 Nov 23;7(6):e00184-22. doi: 10.1128/msphere.00184-22 (PMC9769657; doi:10.1128/msphere.00184-22)
Supplement: TEXT S1 [file msphere.00184-22-s0001.pdf]

S1 APPENDIX: ACTIVE LYSOGENY

Appendix S1. Active lysogeny. Detailed information on the active lysogeny mediated by the FluMu during the experimental evolution.

As mentioned in the main text (see *Results*), the *sdaC* gene suffered stop mutations in three evolved populations bearing pB1000 (Rd/pB-T100). In replicates II and III, the truncation was caused by early stop codons, either by a nucleotide insertion or a single nucleotide polymorphism (S2 Table). However, in replicate I, the gene was half divided due to a recombination event mediated by the prophage FluMu (Fig 3, S1 Fig).

H. influenzae RdKW20 is known to have integrated in its chromosome the complete genome of the prophage called FluMu (or Hin-Mu)[1]. This 34,676 bp prophage is a transposable Mu-like phage, which propagates itself by transposition and integration into the host chromosomes. In this case, a spontaneous replication of FluMu produced its transposition and integration in the middle of *sdaC*, making the duplication of a 36 bp long fragment (3’-AAATAGAAAGAACAAATAAAATTAAGACTAACGGAT-5’) (S1 Fig). Not only it truncates the *sdaC* gen, but it also causes a massive recombination event involving 560,460 bp of the chromosome (NC_000907.1: from nucleotide 1,594,400 to 324,662). This event corresponds to the 30.62% of the chromosome, harboring 461 CDSs, 30 tRNA and 9 rRNA genes.

The recombination event was confirmed by PCR and Sanger sequencing. To examine the phage replication and to check if additional phage genomes could have been excised from the chromosome, we inferred the phage copy number as the ratio of the coverage of FluMu and the chromosome. As expected, the prophage presented around one copy per chromosome in every population (μ = 1.56, SD = 0.34), with the exception of the recombined population (Rd/pB-T100, replicate I), which presented 2.94 copies, twice as

many as the others (Fig 3C). We also inferred the copy number of the truncated *H. influenzae* RdKW20 prophage ϕflu[2]. In this case, the copy number was stable over all the populations ($\mu = 0.87$, SD = 0.05) (S1 Table).

Bibliography

1. Fleischmann RD, Adams MD, White O, et al. Whole-genome random sequencing and assembly of Haemophilus influenzae Rd. *Science*. 1995;269(5223):496-512. doi:10.1126/science.7542800

2. Hendrix RW, Smith MCM, Burns RN, Ford ME, Hatfull GF. Evolutionary relationships among diverse bacteriophages and prophages: All the world's a phage. *Proc Natl Acad Sci*. 1999;96(5):2192-2197. doi:10.1073/pnas.96.5.2192
