# Supplementary material for: Genomics, Transcriptomics, and Metabolomics Reveal That Minimal Modifications in the Host Are Crucial for the Compensatory Evolution of ColE1-Like Plasmids
Source: mSphere. 2022 Nov 23;7(6):e00184-22. doi: 10.1128/msphere.00184-22 (PMC9769657; doi:10.1128/msphere.00184-22)
Supplement: TEXT S2 [file msphere.00184-22-s0002.pdf]

S2 APPENDIX: **METABOLOMICS METHODS**

Appendix S2. Metabolomic analysis. Detailed information regarding the materials and methods of the metabolomic analysis.

CE-TOF/MS analysis procedure. CE-ESI(+)-TOF/MS analysis of samples was performed using a CE System (Agilent 7100) coupled to a TOF/MS system (Agilent 6224). The separation occurred in a fused-silica capillary (length, 70 cm; 50 μm ID, Agilent Technologies, Santa Clara, CA, USA) under normal polarity with a background electrolyte containing 1.0 M formic acid in 10% (v/v) methanol at 20 °C. Sheath liquid (6 µL/min) was methanol:water (1:1, v/v) containing 1.0 mM formic acid with two reference masses (hypoxanthine and (1H,1H,3H-tetrafluoropropoxy) phosphazene HP-0921, from Agilent Technologies, Santa Clara, CA, USA) to allow correction and high mass resolution in the MS. Samples were hydrodynamically injected at 50 mbar for 100 s and stacked by injecting background electrolyte at 100 mbar for 20 s. Then, samples were separated using a capillary voltage of +30 kV, achieving a current of ~2.4 mA.

Analysis of samples was performed under positive ESI mode. The optimized MS parameters were: fragmentor 125 V, skimmer 65 V, octopole 750 V, nebulizer pressure 10 psi drying gas temperature at 200 °C and flow rate 10.0 L/min. The capillary voltage was 5500 V. Data were acquired in full MS scan mode using a mass range of *m/z* 70 to 1000 and an acquisition rate of 1.36 spectra/s. Mass correction was performed using *m/z* 121.0509 and 922.0098.

CE-TOF/MS data processing procedure. Once the samples were analyzed, full scan data collected in ESI+ mode were cleaned and unrelated ions by the Batch Recursive Feature Extraction tool from Agilent MassHunter Workstation Software Profinder B.10.00. Subsequently, potential compounds found by deconvolution were annotated by

comparing their accurate mass, adduct profile and relative migration times with an in-house library of pure standards analyzed under the same method[1]. Unknown molecular features were screened for potential annotations matching the accurate mass using the Batch Search option of the CEU Mass Mediator metabolite annotation tool[2]. Blank subtraction, normalization by internal standard (methionine sulfone, Sigma-Aldrich, Steinheim, Germany), filtering by presence in the QCs (deleted if not present in 100% of the QC samples), filtering by presence in sample groups (deleted if not present in at least 60% of one sample group), and filtering by QC RSD (deleted if showing QC RSDs values higher than 20%). Samples were analyzed in a 'randomized batch order': samples acquired from each evolution line constituted 'batches', whose sequence of analysis was randomized; then, inside each 'batch', the sequence of analysis of Rd-T0, Rd/pB-T0, Rd/pB-T100 and Rd-T100 samples was additionally randomized.

LC-QTOF/MS analysis procedure. Briefly, 2 µL and 5 µL of sample extract were injected for ESI+ and ESI- analyses, respectively, into a thermostated Agilent InfinityLab Poroshell 120 EC-C18 column (3.0 mm × 100 mm, 2.7 µm; Agilent Technologies, Santa Clara, CA, USA) at 50ºC, equipped with an Agilent InfinityLab Poroshell 120 EC-C18 guard column (3.0 mm × 5 mm, 2.7 µm; Agilent Technologies, Santa Clara, CA, USA). The flow rate was 0.6 mL/min with solvent B (10 mM $C_2H_7NO_2$, 0.2 mM NH_4F in 2:3:5 acetonitrile:methanol:isopropanol (v/v)) and solvent A (10 mM $C_2H_7NO_2$, 0.2 mM NH_4F in 9:1 water:methanol (v/v)) for analysis in both positive and negative ionization modes. Initial conditions at time 0 were 70% B, held until 1 min. Next, the percentage of organic phase was gradually increased up to 86% B at 3.5 min, held until 10 min. Subsequently, the composition of the mobile phase was enriched in organic phase until 100 % B at 11 min and held until 17 min. The conditions were then returned to the starting conditions by 17.16 min, followed by a 1.84 min re-equilibration time. Reference mass solution was

5% Milli-Q water in acetonitrile, containing three reference masses to allow correction and high mass resolution in the MS (Agilent Technologies, Santa Clara, CA, USA). The total run time of the method was 19.0 min. The capillary voltage was set to 3500 kV in both polarities; the drying gas flow rate was 12 L/min at 300 °C and the gas nebulizer at 50 psi; fragmentor voltage, skimmer voltage and octopole radio frequency voltage were set to 150, 65 and 750 V, respectively.

Data were collected at a scan rate of 3.5 spectra/s. Mass spectrometry detection was performed in positive and negative ESI modes in a full scan 50-3000 *m/z*. Mass correction was performed using the *m/z* 121.0509 and 922.0098 in positive polarity, and *m/z* 112.9856, 980.0164 and 1033.9881 in negative polarity. Samples were analyzed in separate runs (positive and negative), in a randomized batch order as described for CE-TOF/MS. Subsequently, two MS^2 analyses under each polarity in iterative auto MS^2 were performed under the above-described chromatographic conditions. Tandem mass spectra were massively collected actively excluding previously fragmented precursor ions (isolation width ≈ 1.3 Da) during several injections of the same sample pool. Precursor ions were subjected to collision-induced dissociation, using a ramped collision energy with a slope and offset values of 3.8 and 4.6, respectively.

LC-QTOF/MS data processing procedure. Full scan MS data collected from both ESI+ and ESI- polarity modes were cleaned and unrelated ions by the Batch Recursive Feature Extraction tool using Agilent MassHunter Profinder version B.08.00 software. Data were extracted using a data mining algorithm based on the software and exported as independent spreadsheet files. MS/MS-containing data files were processed using Agilent MassHunter Lipid Annotator 1.0, which renders putatively annotated features with an associated MS^2 spectra. Metabolites annotated in Agilent MassHunter Lipid Annotator output files were matched to compatible molecular features in terms of retention time,

adduct profile and isotopic distribution present in spreadsheet output files generated from MS^1 data described above using Agilent MassHunter Workstation Software Qualitative Analysis B.07.00.

Subsequently, these spreadsheet files were manually revised and screened for potential gaps in compound classes with compatible elution order and adduct profile using the Batch Search of the CEU Mass Mediator metabolite annotation tool[2]. MS^1 data files were screened for subsets of compounds using the Batch Targeted Feature Extraction tool from Agilent MassHunter Workstation Software Profinder B.10.00. Manual curation of compound integration was subsequently performed. Blank subtraction, filtering by presence in the QCs, sample groups, and by QC RSD were applied with the aforementioned criteria.

GC-QTOF/MS analysis procedure. Sample analysis was performed on an Agilent Technologies 7890B GC system coupled to an Agilent Technologies 7250 accurate mass Q/TOF analyzer equipped with an electron ionization (EI) source. 2 µL of sample were placed into a multimode inlet at 250 °C with a 3:1 split ratio, connected to a capillary column (Agilent DB-5MS, 30 m length +10 m guard column, 0.25 mm ID, 0.25 µm film thickness; 95% dimethyl—5% diphenylpolysiloxane; Agilent Technologies, Santa Clara, CA, USA). Helium was used as carrier gas, at a flow rate of 0.85 mL/min. Column temperature was initially set at 60°C for 1 min and then programmed to increase at a rate of 10 °C·min-1 until 325 °C which was maintained for 10 min. Total runtime was 37.5 min. Full MS scan mode was chosen as the acquisition mode, with an acquisition rate of 6.67 spectra/s and an acquisition mass range of 45-650 m/z. Samples were analyzed in a randomized batch order as described for CE-TOF/MS.

GC-QTOF/MS data processing procedure. Sample data files acquired in full scan MS, profile mode, were converted to TDA format. Subsequently, samples were subjected to

automated compound search using the SureMass algorithm from Agilent MassHunter Unknowns Analysis B.09.00 to screen the data for the presence of metabolites[4]. A preliminary search was performed using an in-house PCDL library containing exact mass and retention time of pure standards (740 entries), where minimum match factor value of 80 was selected. Additionally, a second search was performed using the NIST and Fiehn libraries[3] with minimum match factor values of 80 and 50, respectively. Since the NIST library does not contemplate RT as a constraint for scoring annotations, a higher match factor was set for the spectral similarity search to reduce the number of false positives. Retention time and exact mass were considered as match criteria. A fatty acid methyl ester mix (methyl esters mix (C8:0 – C22:1) Ref. CRM47801 (Supelco, Sigma-Aldrich Chemie GmbH, Steinheim, Germany) was used for estimation of Fiehn retention indexes. Subsequently, compound annotations were manually curated, and qualifier and quantifier ions were selected based on abundance and specificity of data. Compound integration was performed using Agilent MassHunter Workstation Software Quantitative analysis for TOF B.09.00. A curated data matrix containing integrated areas for each compound in the different samples was exported. Signals derived from the column bleeding were eliminated. Blank subtraction, normalization by internal standard (tricosane, Sigma-Aldrich, Steinheim, Germany), filtering by presence in the QCs and in sample groups with the aforementioned criteria, and filtering by QC RSD (values higher than 30% were deleted).

Bibliography

1. Mamani-Huanca M, de la Fuente AG, Otero A, et al. Enhancing confidence of metabolite annotation in Capillary Electrophoresis-Mass Spectrometry untargeted

metabolomics with relative migration time and in-source fragmentation. *J Chromatogr A*. 2021;1635:461758. doi:10.1016/j.chroma.2020.461758

2. Gil-de-la-Fuente A, Godzien J, Saugar S, et al. CEU Mass Mediator 3.0: A Metabolite Annotation Tool. *J Proteome Res*. 2019;18(2):797-802. doi:10.1021/acs.jproteome.8b00720

3. Kind T, Wohlgemuth G, Lee DY, et al. FiehnLib: mass spectral and retention index libraries for metabolomics based on quadrupole and time-of-flight gas chromatography/mass spectrometry. *Anal Chem.* 2009;81(24):10038-10048. doi:10.1021/ac9019522

4. Rey-Stolle F, Dudzik D, Gonzalez-Riano C, Fernández-García M, Alonso-Herranz V, Rojo D, Barbas C, García A. Low and high resolution gas chromatography-mass spectrometry for untargeted metabolomics: A tutorial. *Anal Chim Acta* 2021. doi: 10.1016/j.aca.2021.339043
